# The safety of early versus late ileostomy reversal after low anterior rectal resection: a retrospective study in 47 patients

**DOI:** 10.1186/s13037-020-00275-1

**Published:** 2021-01-10

**Authors:** Ian Fukudome, Hiromichi Maeda, Ken Okamoto, Hajime Kuroiwa, Sachi Yamaguchi, Kazune Fujisawa, Mai Shiga, Ken Dabanaka, Michiya Kobayashi, Tsutomu Namikawa, Kazuhiro Hanazaki

**Affiliations:** 1 Department of Surgery, Kochi Medical School, Kohasu, Oko-cho, 783-8505 Nankoku-city, Kochi Japan; 2 Cancer Treatment Center, Kochi Medical School, Kohasu, Oko-cho, Nankoku-city, 783-8505 Kochi Japan; 3 Integrated Center for Advanced Medical Technologies (ICAM-Tech), Kochi Medical School, Nankoku-city, Japan

**Keywords:** Stoma closure, Low anterior resection, Diverting ileostomy

## Abstract

**Background:**

This study aimed to clarify the safety of early closure in diverting ileostomy with lower anterior rectal-cancer resection.

**Methods:**

We retrospectively reviewed consecutive 47 patients who underwent diverting ileostomy with lower rectal-cancer resection between May 2009 and October 2017. The results of the stoma closure were compared between patients who underwent stoma closure within 90 days (early closure [EC] group) and those who underwent late closure (LC group; closure after 90 days). Because of the small sample size, the frequency of severe complications post closure was analyzed.

**Results:**

Among 47 patients, 29 were in the EC group. Postoperative complications occurred in 48.3% (14/29) and 27.8% (5/18) of patients in the EC and LC groups, respectively. This difference was due to minor complications (Clavien-Dindo Classification I/II), such as superficial incisional surgical site infections (*n*=5) in the EC group. The rate of severe complications (Clavien-Dindo Classification ≥ III) was similar between the groups (20.7% vs. 16.7%, *p*=1, Fisher’s exact test).

**Conclusions:**

No association was observed between the time of closure and development of major complications; however, there was an increased likelihood of minor complications after EC. This study provides a basis on which future treatment guidelines for early stoma closure may be developed without affecting patient quality of life.

## Background

Anastomotic leakage is one of the most serious complications after lower anterior resection, with an occurrence rate of 3–28% based on a meta-analysis and literature review [1, 2]. The preventive role of the trans-anal rectal drainage tube is controversial [3–5], and creation of a diverting ileostomy is reliable for the prevention of an anastomotic leakage [6, 7]. Stoma closure is generally scheduled 3–6 months after the initial surgery, when wound healing is uneventful [8, 9]. However, a diverting ileostomy may cause major psychological and physical stress, leading to a decrease in the quality of life [10, 11]; thus, patients often request an earlier reversal.

A few randomized clinical trials have elucidated the optimal timing for reversal of the diverting ileostomy [12, 13]. Although the safety of very early stoma closure (within 2 weeks), compared to conventional stoma closure (after 3 months), was suggested, each of these trials has limitations. A recent trial comparing the safety of closing an ileostomy within 8–13 days after rectal resection and 12 weeks after initial surgery demonstrated a similar rate of severe postoperative complications between the groups [12]. However, the inclusion of 127 of the 418 patients who were assessed for eligibility suggests that this study had strict patient selection criteria. Alves et al. reported that early stoma closure on day 8 after initial surgery was feasible, with reduced hospital stay, bowel obstruction, and medical complications [13]. However, the recruited patients were young, with a mean age of 58 years, and seemed to have clinical characteristics different from those of subjects in our country [14, 15] and institutes [16]. Although colorectal surgery has been proven to be safe in elderly patients [16], repeated surgical interventions within a short period of time must be carefully assessed in patients with poor general health.

Owing to the lack of enough evidence concerning early stoma closure, this retrospective study aimed to evaluate the safety of early closure (EC) (< 90 days after operation) of diverting ileostomy created during lower anterior resection for rectal cancer by reviewing the clinical data of our patients.

## Methods

### Patient groups

The data of consecutive patients (*N* = 50) who underwent lower anterior resection for rectal tumors and diverting ileostomy at the Kochi Medical School Hospital in Japan between May 2009 and October 2017 were collected. After excluding two cases with a simultaneous hepatectomy and one case with failure to close the diverting ileostomy, we analyzed the data of 47 patients. Because stoma closure is often scheduled 3 months after the initial operation, we determined to divide the patients into 2 groups at 90 days. Those who underwent stoma closure within 3 months (within 90 days) were grouped into the EC group, while those who underwent stoma closure after 90 days or more were grouped into the late closure group (LC group). Data regarding complications developed within 1 year of stoma closure were collected. This study was approved by the Institutional Review Board of the Kochi Medical School (ERB-103,996, 30–44), with an opt-out system in place for participant consent.

### Operation and management

Lower anterior resection was performed with total mesorectal excision [17] or tumor-specific mesorectal excision and regional lymphadenectomy. Pelvic lateral lymph node dissection was not routinely performed in our institute. Instead, resection or chemo-radiation was performed for the swollen nodes detected by preoperative imaging tests. End-to-end anastomosis was performed using the double-stapling technique or trans-anal coloanal anastomosis. When anastomosis was performed at the level of the levator ani muscle, a diverting ileostomy was created. Water intake was usually allowed on the first postoperative day, and oral intake of solid food was initiated from the second postoperative day. Typically, we closed the diverting ileostomy within 3–6 months after the initial operation; however, no stipulated guideline currently exists regarding ileostomy closure. For the test of anastomosis, the Barium enema and CT scan before stoma colure was performed the most frequently, while endoscopy was rarely performed.

The procedure for ileostomy closure began with a skin incision made in a spindle-like fashion around the ileostomy. Closure of the intestinal orifice with 1 − 0 silk sutures (1 − 0 Braided Silk, Akiyama-seisakusho. CO., Ltd, Japan) was then performed to maintain a clear operative field. Next, the ileum was circumferentially dissected and mobilized from the abdominal wall. After lifting the stoma out of the wound, a functional end-to-end anastomosis with an automatic suturing device (ETHICON Linear cutters, Johnson and Johnson MEDICAL DEVICES COMPANIES, America) was performed. The peritoneum, posterior layer of the fascia, and anterior layer of the fascia were closed, separately. After washing the subcutaneous tissue with normal saline (OTSUKA NORMAL SALINE, Otsuka, Japan), the skin was closed using 4 − 0 absorbable dermal sutures (4 − 0 Monodiox, Alfresa Pharma Corporation, Japan).

### Statistical analysis

The frequency of the occurrence of postoperative complications (Clavien-Dindo Classification III or more) was compared using Fisher’s exact test. *p* < 0.05 was considered statistically significant. No other analysis or multivariate analysis was planned owing to the small number of included patients. For statistical analysis, EZR version 1.40 was used.

## Results

### Patient characteristics

The mean age of the patients was 66.1 years, and 59.6% patients were male (Table 1). Stoma closure within 90 days (EC group) was performed in 29 (62%) of 47 cases. Most patients had been diagnosed with early rectal cancer, and they were treated with laparoscopic lower anterior resection. Preoperative chemotherapy was provided only in 1 patient in the EC group. Adjuvant chemotherapy was performed in 19% of patients, mainly for Stage III disease.

**Table 1:** Clinical features of the patients

	Total *N*=47	EC group *N*=29	LC group *N*=18
Age			
year, Median [range]	68 [33-86]	69 [33-86]	66.5 [48-79]
Gender			
Male/Female	28/19	15/14	13/5
Tumor location			
Rs/Ra/Rb	1/8/38	1/7/21	0/1/17
Stage			
I	24 (51.1)	16 (55.2)	8 (44.4)
II	8 (17.0)	4 (13.8)	4 (22.2)
III	9 (19.1)	5 (17.2)	4 (22.2)
IV	5 (10.6)	4 (13.8)	1 (5.6)
Other	1 (2.1)	0	1 (5.6)
Approach and surgery			
Lap-LAR	38 (80.9)	22 (75.9)	16 (88.9)
Open-LAR	4 (8.5)	4 (13.8)	0
Lap-LAR(Trans-anal)	3 (6.4)	3 (10.3)	0
Open-LAR(Trans-anal)	2 (4.3)	0	2 (11.1)
Adjuvant chemotherapy			
Yes (%)	9 (19.2)	3 (10.3)	6 (33.3)
Neoadjuvant chemotherapy			
Yes (%)	1 (2.1)	1 (3.5)	0 (0)
Interval between creation and closure of stoma			
day, Median [range]	79 [15-371]	58 [15-90]	107.5 [91-371]

*Lap-* laparoscopic assisted, *LAR* low anterior resection, *Trans-anal* trans-anal anastomosis

The mean (median) interval time between initial surgery and stoma closure was 94.9 days [range; 15–371 days]. The mean time to stoma closure was 57.2 days (range: 15–90 days) and 156.8 days (range: 91–371 days) in the EC and LC groups, respectively.

### Postoperative complications after initial treatment

Postoperative complications developed in 23 (49%) of 47 patients; however, there were no surgery-related deaths (Table 2). Anastomotic leakage was observed in 4 patients (9%). In EC group, 24% of the patients suffered from the complications related to the ileostomy, such as obstruction of the jejunum when it passed through the abdominal wall, internal hernia of oral-side small intestine related to the stoma, and ileus due to adhesion of the jejunum to the abdominal wall around the stoma. Meanwhile, 5.5% (1/18) of patients in the LC group developed these complications. Therefore, we found that stoma-related complications that were refractory to conservative treatment often resulted in early stoma closure (Table 2).

**Table 2:** Postoperative complications occurring between initial operation and stoma closure

	*N*=47	EC group *N*=29	LC group *N*=18
The number of patients with postoperative complications			
	23 (48.9)	14 (48.3)	9 (50.0)
Incidence of the complications			
Anastomotic leakage	4 (8.5)	2 (6.9)	2 (11.1)
Ileostomy-related ileus	8 (17.0)	7 (24.1)	1 (5.6)
Other intestinal obstruction	6 (12.8)	3 (10.3)	3 (16.7)
Abdominal abscess	0	0	0
Other complications^a^	5 (10.6)	2 (6.9)	3 (16.7)

The number in the parenthesis is percentage

^a^Other complications included allergic reaction against drug used perioperative period, anemia without bleeding, anxiety after surgery, and recto-bladder fistula

### Complications after stoma closure

**Fig. 1:**
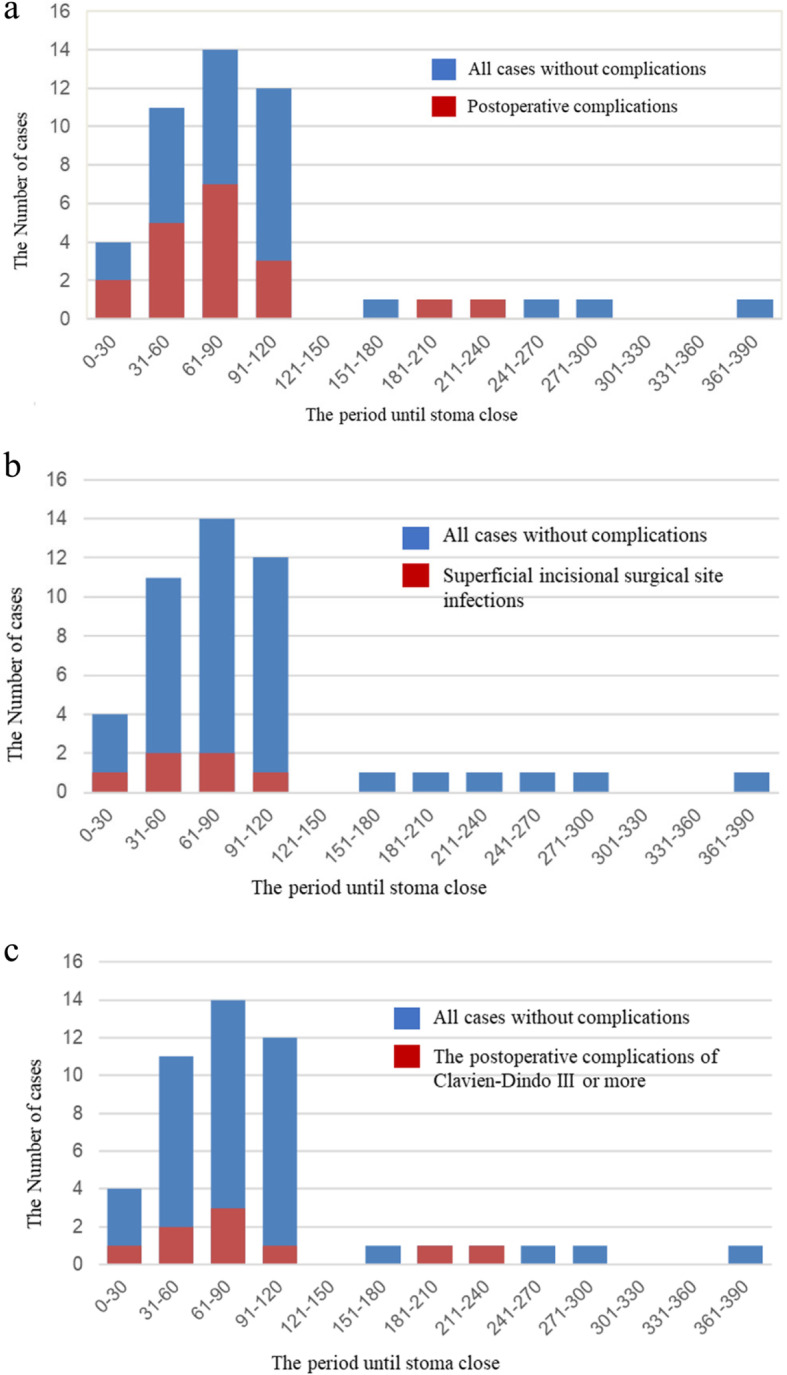
The interval between stoma creation and closure and the occurrence of postoperative complications. Bar graphs show the number of stoma closures per period (30 days) after the initial operation. The number of postoperative complications among the cases is shown in red (**a**) All cases and postoperative complications are demonstrated. The postoperative complications accumulate within 90 days. In the early closure (EC) group, the complications seem to be equally distributed. **b** The accumulation of superficial incisional surgical site infections among patients who underwent stoma closure within 90 days. **c** Postoperative Clavien-Dindo Classification of III or higher complications are highlighted in red

Overall, the rate of postoperative complications was 48.3% and 27.8% in the EC and LC groups, respectively (Table 3; Fig. 1). Within the EC group, the complications seemed equally distributed over 30 days (Fig. 1a). In terms of Clavien-Dindo Classification I/II complications, the rate was 27.6% and 11.2% in the EC and LC groups, respectively. We found that superficial incisional surgical site infections (SSI) developed in 5 and 1 patients in the EC and LC groups, respectively (Fig. 1b). However, the rate of Clavien-Dindo Classification III or higher complications was similar between the groups: 20.7% and 16.7% in the EC and LC groups, respectively (*p* = 1, Fisher’s exact test) (Fig. 1c). These complications included colo-rectal/colo-anal anastomotic stenosis, urinary retention necessitating catheter placement, abscess, ileus, abdominal incisional hernia, and recto-vaginal fistula. Anastomotic leakage of the colorectal anastomosis was not observed. When only the complications within 30-days after operation are counted, the occurrence rate of complications with Clavien-Dindo Classification III or higher is 13.7% (4/29) for EC group and 5% (1/18) for LC group respectively, which has no statistical difference.

**Table 3:** Postoperative complications after stoma closure within 1 year

	EC group *N*=29	LC group *N*=18
All complications		
	14 (48.3)	5 (27.8)
Calvien-Dindo classification		
1	4 (13.8)	1 (5.6)
2	4 (13.8)	1 (5.6)
3a	4 (13.8)	0
3b	2 (6.9)	3 (16.7)
Calvien-Dindo classification ≥ 3*	6 (20.7)	3 (16.7)
Type of complications		
Anastomotic leakage (colo-anal)	0	0
Abdominal abscess	1 (3.4)	0
Wound infection of abdominal wall	5 (17.2)	1 (5.6)
Others	8 (27.6)	4 (22.2)
Anastomotic stenosis	1 (3.4)	1 (5.6)
Recto-vaginal fistula	0	1 (5.6)
Abdominal hernia	0	1 (5.6)
Bowel obstruction	2 (6.9)	0
Urinary retention	1 (3.4)	0
Cellulitis at the drop site	1 (3.4)	0
Prolonged fever	2 (6.9)	0
Diarrhea	1 (3.4)	1 (5.6)

Statistical comparison was performed only to compare the frequency of complication with Calvien-Dindo classification ≥ 3*, because the number of the included patients are limited in the present study. The data analysis didn7t reveal the significant difference between two groups (Fisher’s exact test, *P*=1)

## Discussion

This retrospective study showed no association between the time of closure and development of major complications. However, EC is likely associated with higher occurrence of minor complications. This study is uniquely valuable because of the heterogeneous characteristics of the patients, including a wide age range, the Eastern Cooperative Oncology Group Performance Status Scale (ECOG-PS), and postoperative complications after initial surgery [12, 13]. Furthermore, the study demonstrated two intriguing results.

First, early stoma closure within 90 days after the initial operation was not associated with seriouscomplications such as leakage of the colorectal/coloanal anastomosis, or abdominal abscess formation. The total complication rate of Clavien-Dindo Classification III or more was 20.7% and 16.7% in the EC and LC groups (Table 3), respectively, suggesting no significant difference between the groups. Thus, we can assume that early stoma closure is a feasible management option when preoperative radiological examinations suggest no anastomotic leakage. However, one concern is that the frequency of severe postoperative complications (Clavien-Dindo Classification ≥ III) in the study is greater than that reported in previous studies [12, 13]. Danielsen reported a complication rate of 3.6% and 7% in the EC and LC groups, respectively [12]. Inclusion of old cases and a longer observation period after stoma closure may partially explain the high incidence of postoperative complications in our study. However, further improvement in the high rate of postoperative complications in our institute is needed.

The next clinical question to be answered is, what is the best timing for stoma closure within 90 days? A clinical trial demonstrated that stoma closure at 30 days after the initial operation increased the rate of severe postoperative complications when compared with that of late stoma closure [18]. Although the authors could not explain the reasons for their results, adhesion at 30 days after the initial operation could be worse than after a longer period, and difficulties during the operation might result in higher occurrence of severe postoperative complications. In the present study, only three patients underwent stoma reversal 20–40 days after the initial surgery. However, we should note that one of these patients developed a pelvic abscess after stoma closure. Further studies should focus on analyzing the safety of stoma closure approximately 1 month after the initial operation. We believe that elective stoma closures within 1 month should be avoided until clear evidence of their safety has been established.

The second intriguing point is the possibility of an increased incidence in minor postoperative complications, Clavien-Dindo Classification I/II, after early stoma closure. For instance, superficial incisional SSI was observed in 17.2% (5/29) and 5.6% (1/18) of patients after early and delayed closure, respectively (Table 3). One explanation for this could be that early stoma closure in our patients was performed when stoma-related complications could not be conservatively resolved rather than when the postoperative course was uneventful. Therefore, patients who underwent early stoma closure could have had a worse general condition, or local inflammation around the stoma, leading to the high incidence of minor postoperative complications. Our institution started to adopt a purse-string closure technique, which is associated with significantly fewer SSIs and better cosmetic outcomes with stoma reversal than those associated with conventional primary closure [19], thus potentially reducing the postoperative wound infection rate even after early stoma closure. Furthermore, applying anti-adhesive materials [20] around the stoma could reduce the technical difficulties experienced and further improve the results of early stoma closure.

The limitations of this study are the small number of patients and the unadjusted comparison between the two groups. Because the occurrence of postoperative complications is multi-factorial, a simple comparison may exaggerate or mask the differences. Thus, we focused on the statistical comparison of severe complications, resulting in the obscurity of other complications. Further, we could not assess the safety of “very” early stoma closure, within 2 weeks of the initial surgery. Although clinical trials have suggested the safety of very early stoma closure in selected patients, further studies with the inclusion of older patients are necessary before recommending early stoma closure for all patients. Furthermore, a small number of patients who underwent preoperative chemotherapy (or chemo-radiation) were included in this study. Hence, our results cannot be extrapolated in these clinical settings because of the potential risks associated with early stoma closure due to the poor clinical condition of these patients [21].

We are currently conducting and awaiting the results of a clinical trial investigating whether very early stoma closure within 2 weeks of initial surgery is safe. (UMIN ID: 000036382, registered on 03/04/2019).

## Conclusions

This study did not suggest that early stoma closure performed within 90 days after initial surgery increased the frequency of life-threatening, severe postoperative complications, even in vulnerable patients such as the elderly. However, the safety of early stoma reversal within 2 weeks after the initial operation, which is often discussed in the literature, was not answered from the present study. The results of this study can be used to develop clinical trials that reflect the outcomes of early stoma closure in the general population and may be used to develop future treatment guidelines.

## Data Availability

Not applicable.

## Abbreviations

EC: Early closure
LC: Late closure
SSI: Surgical site infection
ECOG-PS: Eastern Cooperative Oncology Group Performance Status Scale
